# Thrombophilia and Folate Cycle Gene Polymorphisms in the Development of Ischemic Stroke After COVID-19

**DOI:** 10.3390/ijms27062650

**Published:** 2026-03-13

**Authors:** Dildora Khaydarova Kadirovna, Nodirjon Kadirovich Khaydarov, Sanobar Nizamovna Rakhmatova, Nilufar Kahhorovna Salomova, Visola Furkatovna Gaffarova, Qunduz Abdullo Qizi Sadulloyeva, Dilshod Izbilloyevich Sadullayev, Mukhammadjon Kahramon Ugli Berdiyev, Bakhodir Igamovich Djumayev, Nodirabegim Akbarovna Shukrulloeva, Ferangiz Shuxratovna Mukhamadieva, Ibodov Bekzod Abdusattotovich, Dilbar Tadjievna Khodjieva

**Affiliations:** 1 Department of Neurology and Medical Psychology, Tashkent State Medical University, Farabi Street 2, Tashkent 100109, Uzbekistan; dildora_doktor@mail.ru (D.K.K.); prof.xaydarov@gmail.com (N.K.K.); doctorbek.707@mail.ru (I.B.A.); 2 Department of Neurology, Bukhara State Medical Institute, Gijduvan Street, 23, Bukhara 100100, Uzbekistan; sanobarnevro76@gmail.com (S.N.R.); nilufarsalomova535@gmail.com (N.K.S.); visolagafforova7@gmail.com (V.F.G.); sadullayevaq1998@gmail.com (Q.A.Q.S.); sadullaev_2021@mail.ru (D.I.S.); muhammadjonberdiyev@gmail.com (M.K.U.B.); djumayevbahodir778@gmail.com (B.I.D.); nodirabegim121@gmail.com (N.A.S.); fera19941204@gmail.com (F.S.M.)

**Keywords:** COVID-19, ischemic stroke, thrombophilia, folate cycle gene polymorphisms, hypercoagulation and inflammation

## Abstract

COVID-19 not only affects the respiratory system but also increases the risk of cerebrovascular complications, including ischemic stroke. Experimental and clinical data suggest that cytokine dysregulation and polymorphisms of thrombophilia-related genes (MTHFR, MTR, and MTRR) may jointly promote hypercoagulation, endothelial dysfunction, and thromboinflammation, thereby contributing to post-COVID ischemic stroke. This study included 160 patients treated at Zangiota Infectious Diseases Hospitals (2021–2023): 60 patients with ischemic stroke in the acute or post-COVID period (experiment group), 50 COVID-19 patients without ischemic stroke (comparison group), and 50 ischemic stroke patients without COVID-19 (control group). Clinical–neurological and immunological parameters were assessed, and polymorphisms in thrombophilia/folate cycle genes (MTHFR C677T, MTR, and MTRR) were genotyped by PCR/real-time PCR. Statistical analysis included χ^2^ tests, *t*-tests, logistic regression with odds ratios (OR) and 95% confidence intervals (CI); Hardy–Weinberg equilibrium was verified. A strong association was identified between the MTHFR C677T polymorphism and ischemic stroke on the background of COVID-19 (OR = 5.4; 95% CI: 2.1–13.8; *p* < 0.001). The TNF-α rs1800629 polymorphism was also significantly associated with COVID-19-related cerebrovascular events (OR = 3.27; 95% CI: 1.4–7.6; *p* = 0.006). Carriage of two or more minor alleles produced a synergistic effect, markedly increasing the risk of post-COVID ischemic stroke (OR = 5.59; 95% CI: 2.3–13.6; *p* < 0.001). These polymorphisms were linked to hyperhomocysteinemia, endothelial dysfunction, and mechanisms contributing to multifactorial arterial ischemic events. The combined assessment of thrombophilia and folate cycle-related genotypes and clinical indicators may provide a potential framework for improved risk stratification. Polymorphisms in MTHFR may appear to represent important genetic determinants of ischemic stroke following COVID-19, particularly in the context of arterial ischemic mechanisms.

## 1. Introduction

Coronavirus disease 2019 (COVID-19), caused by severe acute respiratory syndrome coronavirus 2 (SARS-CoV-2), has emerged as a major global health problem with a wide spectrum of systemic complications that extend far beyond the respiratory tract. Clinical and epidemiological studies have shown that patients with COVID-19 have a higher incidence of ischemic stroke both in the acute phase and in the post-infectious period, with reported rates of approximately 0.1–6.9% and an overall several-fold increase in stroke risk compared with non-infected populations [1,2,3]. The mechanisms underlying COVID-19-related ischemic stroke are multifactorial and involve dysregulated inflammation, endothelial injury, coagulopathy, and direct neurotropic effects of the virus [4,5].

A central element in this process is the COVID-19-induced “cytokine storm,” characterized by excessive release of pro-inflammatory mediators, such as interleukin-1β (IL-1β), interleukin-6 (IL-6), tumor necrosis factor-α (TNF-α), chemokines, and angiogenic factors, including vascular endothelial growth factor A (VEGFA). Hyperproduction of these cytokines promotes leukocyte activation and migration, endothelial dysfunction, increased vascular permeability, platelet activation, and a shift toward a prothrombotic state. Concomitant disturbances of the renin-angiotensin-aldosterone system due to SARS-CoV-2 binding to angiotensin-converting enzyme 2 (ACE2) further enhance vasoconstriction, oxidative stress, and microvascular thrombosis, thereby amplifying the risk of cerebral ischemia [6,7,8]. In parallel, neuropathological and experimental data indicate that SARS-CoV-2 can affect the neurovascular unit directly via neurogenic (e.g., olfactory) and hematogenous routes, leading to blood-brain barrier disruption, microglial activation, and microischemic lesions [9,10].

There is growing evidence that inter-individual variability in the inflammatory and thrombotic response to SARS-CoV-2 is at least partly determined by genetic factors. Polymorphisms in cytokine-related genes, including IL1B, IL6, TNFA, and IL10, have been associated with differences in cytokine expression, stroke susceptibility, and clinical outcome in non-COVID populations [11,12]. Pro-inflammatory variants (e.g., IL1B -511C/T, IL6 -174G/C, and TNFA -308G/A) tend to increase the risk of ischemic events, whereas anti-inflammatory IL10 variants may exert a protective effect [13,14,15]. In addition, polymorphisms in folate-cycle and thrombophilia-related genes, such as MTHFR, MTR, and MTRR, can impair homocysteine metabolism, promote hyperhomocysteinemia, endothelial dysfunction, and atherothrombosis, and have been consistently linked to elevated stroke risk in different ethnic groups [16,17]. The coexistence of several risk alleles in these pathways may have cumulative or synergistic effects on vascular risk.

In the context of COVID-19, the convergence of a systemic cytokine storm with an underlying prothrombotic genetic background may critically modulate the likelihood and severity of ischemic stroke. However, data on the combined impact of cytokine profiles and thrombophilia-related gene polymorphisms on post-COVID ischemic stroke are still limited, particularly in Central Asian populations. Therefore, this study aimed to investigate the relationship and polymorphisms in thrombophilia- and folate-cycle-related genes (MTHFR, MTR, and MTRR) in patients with ischemic stroke after COVID-19 infection and to assess their contribution to the pathogenesis and individual risk of COVID-19-associated ischemic stroke.

## 2. Results

The frequency and distribution of comorbidities were analyzed based on the results of anamnestic and objective examinations conducted in all subjects. The results of the analysis showed that the most common comorbidities were arterial hypertension and cardiovascular diseases. Arterial hypertension was noted in 31.67% (*n* = 19) cases in the experiment group, 44.0% (*n* = 22) in the comparison group, and 12.0% (*n* = 6) in the control group. Cardiovascular diseases were found in 18.3% (*n* = 11), 18.0% (*n* = 9), and 2.0% (*n* = 1), respectively. Obesity had the highest rate in the comparison group (24.0%), 11.67% in the experiment group, and 4.0% in the control group. Diabetes mellitus (type II) was detected at a frequency of 11.67% in the experiment group, 8.0% in the comparison group, and 10.0% in the control group. Other comorbidities were relatively rare. Gastrointestinal diseases were recorded in 6.0% of cases in the control group and 1.67% in the experiment group. Allergic diseases were detected at a frequency of 8.0% in the control group, 4.0% in the comparison group, and 1.67% in the experiment group. When comparing the distribution of comorbidities in different groups, it was found that cardiovascular diseases and arterial hypertension were statistically significantly (*p* < 0.05) more common in the experiment and comparison groups than in the control group. These results confirm that the aforementioned cardiovascular pathologies play an important role as major risk factors in the development of ischemic stroke associated with COVID-19.

Molecular-genetic analysis focused on polymorphisms of genes involved in the folate cycle and associated with a thrombophilic phenotype, including MTHFR rs1801133 (C677T) and rs1801131 (A1298C), MTR rs1805087 (A2756G), and MTRR rs1801394 (A66G). These polymorphisms affect protein function through distinct molecular mechanisms. The rs1801133, rs1801131, and rs1805087 polymorphisms are located within coding regions and result in single amino acid substitutions, leading to reduced catalytic activity of the encoded enzymes. In contrast, the rs1801394 polymorphism, located in the promoter region of the MTRR gene, negatively affects gene expression and reduces functional protein activity. Decreased activity of MTHFR, MTR, and MTRR impairs homocysteine remethylation to methionine, leading to hyperhomocysteinemia. Elevated homocysteine levels promote thrombosis through endothelial dysfunction, increased oxidative stress, imbalance between pro-coagulant and anticoagulant factors, and enhanced thrombus formation, thereby increasing the risk of ischemia in organs and tissues, including the brain. In addition, reduced MTR and MTRR activity may disrupt methylation processes essential for DNA synthesis, repair, and neurotransmitter metabolism, contributing to vascular and neurological pathology. Based on these mechanisms, the above polymorphisms were analyzed in patients with ischemic stroke associated with COVID-19, patients with ischemic stroke unrelated to COVID-19, and healthy controls. The frequency of minor alleles for all studied polymorphisms was highest in the experimental group and lowest in the control group, suggesting that these genetic variants may act as risk factors for ischemic stroke, particularly in the context of COVID-19. Genotype distribution analysis demonstrated that wild-type homozygous genotypes predominated in the control group, whereas heterozygous and mutant homozygous genotypes were more frequent in the experimental group. Specifically, the mutant TT genotype of MTHFR rs1801133 occurred in 10.0% of the experimental group, 4.0% of the comparison group, and 2.0% of controls. The mutant CC genotype of MTHFR rs1801131 was detected in 6.7% of the experimental group and 2.0% of the comparison group but was absent in controls. The mutant GG genotype of MTR rs1805087 was found in 5.0% and 4.0% of the experimental and comparison groups, respectively, and was not detected in the control group. The MTRR rs1801394 GG genotype was rare in both patient groups (1.7–2.0%) and absent in controls. Hardy–Weinberg equilibrium (HWE) analysis demonstrated that genotype distributions for all examined polymorphisms in the experimental, comparison, and control groups did not deviate significantly from expected values (χ^2^ < 3.84, *p* > 0.05). In the control group, observed genotype frequencies closely matched theoretical expectations for all polymorphisms (*p* = 0.54–0.71), confirming genetic equilibrium, representativeness of the control population, and methodological reliability.

Comparative molecular-genetic analysis between patients with COVID-19-associated ischemic stroke and COVID-19-infected controls without stroke demonstrated that polymorphisms of folate cycle-related genes contribute to increased genetic susceptibility to ischemic stroke in the context of COVID-19.

Analysis of Supplementary Table S1 revealed a strong association between the MTHFR rs1801133 polymorphism and COVID-19-related ischemic stroke. The minor T allele was significantly more frequent in the experimental group and was associated with a 4.05-fold increased risk of ischemic stroke (χ^2^ = 15.3, *p* < 0.001; OR = 4.05, 95% CI: 1.94–8.42), whereas the wild-type C allele exerted a protective effect, reducing risk by approximately 75%. Genotype analysis showed that the wild-type homozygous CC genotype significantly reduced stroke risk, while the heterozygous CT genotype significantly increased susceptibility. Although the TT genotype did not reach statistical significance, a clear trend toward increased risk was observed. Similarly, the MTHFR rs1801131 polymorphism demonstrated a statistically significant association with COVID-19-related ischemic stroke. The wild-type A allele showed a protective effect, whereas the minor C allele increased disease risk. The AA genotype was associated with a significantly reduced risk of stroke, while heterozygous and mutant homozygous genotypes showed non-significant trends toward increased susceptibility. For the MTR rs1805087 polymorphism, allele and genotype distributions differed significantly between the experimental and control groups. The minor G allele was associated with a substantially increased risk of ischemic stroke, whereas the AA genotype exerted a protective effect. In contrast, analysis of the MTRR rs1801394 polymorphism revealed no statistically significant association with COVID-19-related ischemic stroke, suggesting a limited pathogenic role in this context. To assess specificity, polymorphism distributions were also compared between patients with COVID-19-associated ischemic stroke and those with ischemic stroke independent of COVID-19 (Supplementary Table S2). Only the MTHFR rs1801133 polymorphism demonstrated a significant difference between these groups. Carriers of the minor T allele had a 2.27-fold increased risk of ischemic stroke following COVID-19 infection, whereas no significant differences were observed for MTHFR rs1801131, MTR rs1805087, or MTRR rs1801394, indicating that these variants are not specific markers of COVID-19-related stroke.

Notably, comparison between patients with COVID-19-independent ischemic stroke and controls showed no statistically significant association for MTHFR rs1801133, further supporting its role as a specific genetic marker for ischemic stroke associated with COVID-19 rather than stroke of other etiologies.

Finally, Hardy–Weinberg equilibrium analysis confirmed genetic equilibrium in all groups (*p* > 0.05), validating the representativeness of the study population and the reliability of genotyping results. Evaluation of predictive efficacy demonstrated that MTHFR rs1801133 had the highest prognostic value, both independently and in combination with other folate cycle gene polymorphisms, highlighting its importance in the pathogenesis and risk stratification of COVID-19-associated ischemic stroke.

Minor alleles of the investigated folate cycle gene polymorphisms were analyzed to assess their predictive efficacy (AUC) for the development of COVID-19-related ischemic stroke and COVID-19-independent ischemic stroke, with sensitivity (SE) and specificity (SP) evaluated in both patient groups. For the MTHFR rs1801133 (C677T) polymorphism, the minor T allele demonstrated moderate predictive performance in the experimental group (AUC = 0.586, SE = 0.33, SP = 0.98), whereas lower predictive accuracy was observed in the comparison group (AUC = 0.535). The MTHFR rs1801131 (A1298C) minor C allele showed comparable but modest predictive efficacy in both groups (AUC ≈ 0.54). Similarly, the MTR rs1805087 (A2756G) minor G allele exhibited moderate predictive value in both COVID-19-related and COVID-19-independent ischemic stroke (AUC = 0.53–0.59). In contrast, the MTRR rs1801394 (A66G) minor allele demonstrated poor predictive performance (AUC < 0.52). Overall, the prognostic models based on minor alleles showed moderate quality (AUC 0.5–0.7) for MTHFR rs1801133, MTHFR rs1801131, and MTR rs1805087, while the MTHFR rs1801133 polymorphism demonstrated superior predictive value specifically for COVID-19-related ischemic stroke, supporting its role as a disease-specific genetic marker. Homozygous non-wild-type genotypes associated with increased disease risk were further evaluated. The TT genotype of MTHFR rs1801133 demonstrated moderate predictive efficacy for ischemic stroke (AUC ≈ 0.50–0.51) with high specificity but low sensitivity. In contrast, homozygous mutant genotypes of MTHFR rs1801131, MTR rs1805087, and MTRR rs1801394 showed unsatisfactory predictive performance (AUC < 0.50) in both patient groups.

These findings indicate that genotype-based models provide limited prognostic accuracy, with meaningful predictive value observed mainly for MTHFR rs1801133. The combined (syntropic) effect of multiple minor alleles was also assessed. Carriers of four minor alleles were detected exclusively in the experimental group (1.7%), whereas three minor alleles were more frequent in the experimental group (8.3%) than in the comparison group (2.0%). The proportion of subjects carrying two minor alleles was highest in the experimental group (38.3%), followed by the comparison (30.0%) and control (10.0%) groups. These results suggest that the accumulation of multiple minor alleles enhances genetic susceptibility, particularly in COVID-19-associated ischemic stroke, supporting a polygenic and synergistic mechanism in disease pathogenesis.

The proportion of subjects carrying double, triple, and quadruple minor alleles of folate cycle gene polymorphisms differed markedly among the experimental, comparison, and control groups. The pathogenetic and prognostic significance of allele-allele (syntropic) interactions was evaluated for both COVID-19-related and COVID-19-independent ischemic stroke and further illustrated using ROC analysis. Carriers of three minor alleles demonstrated limited predictive performance for ischemic stroke, with low sensitivity despite high specificity in both patient groups (AUC ≈ 0.50–0.51). In contrast, carriers of two minor alleles showed substantially better predictive characteristics. In the experimental group, predictive efficacy reached AUC = 0.62, with sensitivity of 0.38 and specificity of 0.90, while in the comparison group, predictive efficacy was AUC = 0.60, with sensitivity of 0.30 and specificity of 0.90. Importantly, subjects carrying two minor alleles exhibited a 5.59-fold increased risk of developing COVID-19-related ischemic stroke (95% CI: 1.94–16.155; *p* < 0.001), whereas the risk of COVID-19-independent ischemic stroke was increased by 3.86-fold (95% CI: 1.27–11.64; *p* = 0.01). According to the simplified predictive model, the estimated probability of ischemic stroke reached 63% in COVID-19-infected patients carrying two minor alleles, compared with approximately 60% in patients without COVID-19.

These findings indicate that the accumulation of two minor alleles in folate cycle gene polymorphisms confers a markedly increased risk of ischemic stroke, with a stronger effect observed in the presence of COVID-19, supporting a synergistic polygenic mechanism underlying disease susceptibility.

## 3. Discussion

**Figure 1:**
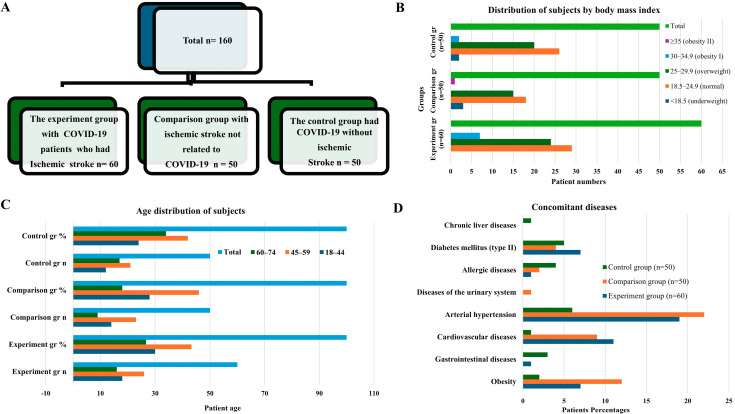
Study design and baseline characteristics of the study population. (**A**) Flow diagram of patient enrollment and group allocation. A total of 160 subjects were included: the experimental group comprised patients with ischemic stroke associated with COVID-19 (*n* = 60); the comparison group included patients with ischemic stroke not related to COVID-19 (*n* = 50); and the control group consisted of patients with COVID-19 without ischemic stroke (*n* = 50). (**B**) Distribution of subjects according to body mass index (BMI) categories in the experimental, comparison, and control groups, including normal weight, overweight, obesity class I (BMI 30–34.9 kg/m^2^), and obesity class II (BMI ≥ 35 kg/m^2^). (**C**) Age distribution of subjects across the three study groups, stratified into age categories 18–44, 45–59, and 60–74 years, presented as absolute numbers and percentages. (**D**) Prevalence of concomitant diseases in the experimental, comparison, and control groups, including arterial hypertension, cardiovascular diseases, diabetes mellitus type II, obesity, gastrointestinal diseases, allergic diseases, urinary system diseases, and chronic liver diseases.

**Figure 2:**
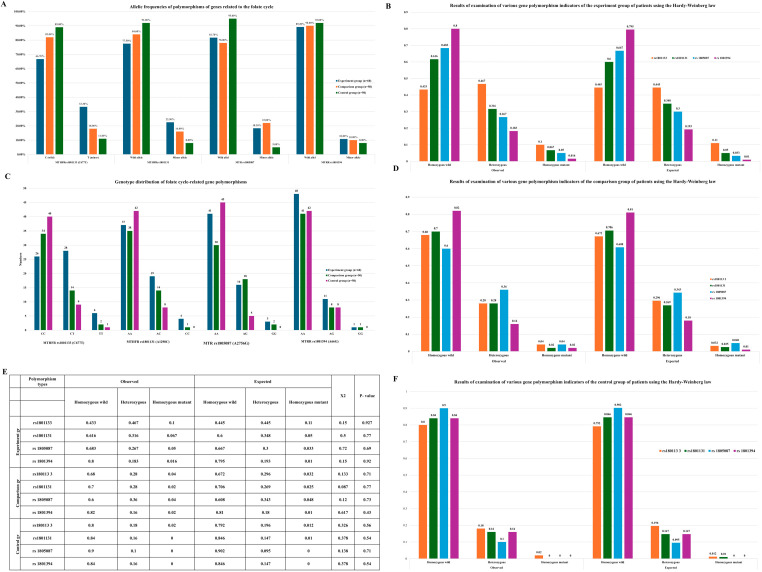
Allelic and genotypic distribution of folate cycle-related gene polymorphisms and Hardy–Weinberg equilibrium analysis. (**A**) Allele frequencies of polymorphisms in folate cycle-related genes (MTHFR rs1801133 (C677T), MTHFR rs1801131 (A1298C), MTR rs1805087 (A2756G), and MTRR rs1801394 (A66G)) in the experimental, comparison, and control groups. Data are presented as percentages of wild-type and minor alleles. (**B**) Observed and expected genotype frequencies of folate cycle-related gene polymorphisms in the experimental group, assessed according to the Hardy–Weinberg equilibrium (HWE). (**C**) Genotype distribution of folate cycle-related gene polymorphisms across the experimental, comparison, and control groups, expressed as absolute numbers of individuals for each genotype. (**D**) Observed and expected genotype frequencies of folate cycle-related gene polymorphisms in the comparison group, evaluated using the Hardy–Weinberg law. (**E**) Summary table of Hardy–Weinberg equilibrium analysis for all studied polymorphisms in the experimental, comparison, and control groups, including observed and expected genotype frequencies, χ^2^ values, and corresponding *p*-values. (**F**) Observed and expected genotype frequencies of folate cycle-related gene polymorphisms in the control group, assessed according to the Hardy–Weinberg equilibrium.

**Figure 3:**
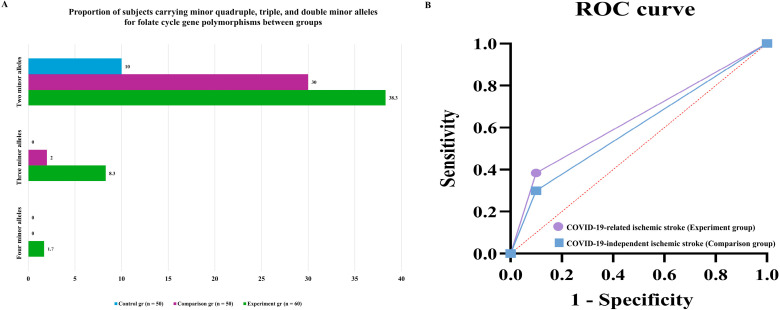
Predictive significance of multiple minor alleles of folate cycle gene polymorphisms in ischemic stroke. (**A**) Distribution of subjects carrying two, three, or four minor alleles of folate cycle-related gene polymorphisms in the experimental (COVID-19-related ischemic stroke), comparison (COVID-19-independent ischemic stroke), and control groups. (**B**) Receiver operating characteristic (ROC) curves illustrating the predictive performance of carrying two minor alleles for the development of ischemic stroke in the experimental and comparison groups.

The present study demonstrates that polymorphisms of folate cycle-related genes are significantly associated with the development of ischemic stroke, with distinct genetic patterns observed for COVID-19-related and COVID-19-independent disease [18]. Among the analyzed variants, MTHFR rs1801133 (C677T) emerged as the most relevant and specific genetic marker for ischemic stroke in the setting of COVID-19. Baseline clinical characteristics, including age distribution, body mass index, and comorbid conditions, were comparable across the study groups, supporting the validity of genetic comparisons (Figure 1) [19]. Analysis of allele frequencies revealed that minor alleles of folate cycle genes were consistently more frequent in the experimental group than in the comparison and control groups (Figure 2A), while genotype distributions followed Hardy–Weinberg equilibrium in all groups (Figure 2B–F; Table 1). A statistically significant association was observed between COVID-19-related ischemic stroke and polymorphisms of MTHFR rs1801133, MTHFR rs1801131, and MTR rs1805087 (χ^2^ > 3.84; *p* < 0.05). However, among these variants, only the minor T allele of MTHFR rs1801133 demonstrated a strong and specific effect in patients with COVID-19, increasing the risk of ischemic stroke by 4.05-fold (95% CI: 1.94–8.42; χ^2^ = 15.3; *p* < 0.001; Table 1; Supplementary Table S1). In contrast, the minor alleles of MTHFR rs1801131 (A1298C) and MTR rs1805087 (A2756G) increased the risk of ischemic stroke in both COVID-19-related and COVID-19-independent contexts (Table 1), indicating a non-specific thrombophilic effect rather than COVID-19 specificity. Predictive performance analysis further supported this distinction. The MTHFR rs1801133 minor allele showed a higher predictive value for COVID-19-related ischemic stroke than for COVID-19-independent ischemic stroke (AUC = 0.586 vs. 0.535; Table 1), whereas the MTR rs1805087 minor allele demonstrated greater predictive efficiency for COVID-19-independent stroke (AUC = 0.585 vs. 0.531). These findings indicate that while multiple folate cycle polymorphisms contribute to ischemic stroke susceptibility, MTHFR rs1801133 uniquely interacts with COVID-19-related pathophysiological mechanisms. The biological plausibility of these results is supported by the functional consequences of the C677T polymorphism, which reduces MTHFR enzymatic activity and promotes hyperhomocysteinemia. Elevated homocysteine levels induce endothelial dysfunction, oxidative stress, and a pro-thrombotic state, processes that are further amplified by COVID-19-associated endothelial injury, inflammation, and coagulation dysregulation [20]. This synergistic interaction likely explains the specificity of MTHFR rs1801133 for COVID-19-related ischemic stroke observed in our study. Importantly, analysis of syntropic (allele-allele) effects demonstrated that accumulation of minor alleles markedly increased disease risk. Carriers of two minor alleles exhibited a 5.59-fold increased risk of COVID-19-related ischemic stroke (95% CI: 1.94–16.155; *p* < 0.001), compared with a 3.86-fold increase for COVID-19-independent ischemic stroke (95% CI: 1.27–11.64; *p* = 0.01; Figure 3A,B; Table 1) [21,22,23]. Consistently, predictive performance was higher in the COVID-19 group (AUC = 0.63 vs. 0.60; Figure 3B); findings highlight the role of polygenic interactions in modulating susceptibility to ischemic stroke in the setting of COVID-19 infection. The observed genetic associations suggest that COVID-19-related ischemic stroke may involve a distinct molecular background compared with ischemic stroke of other etiologies. In this framework, polymorphisms in folate cycle-related genes should be considered primarily as modifiers of arterial ischemic risk. Among the analyzed variants, MTHFR rs1801133 emerges as a potential genetic factor specifically associated with ischemic stroke occurring during COVID-19. In contrast, polymorphisms in MTR and MTRR may contribute more broadly to ischemic stroke susceptibility through mechanisms related to impaired homocysteine metabolism, endothelial dysfunction, and vascular inflammation. Clinically, the presence of combined risk genotypes, particularly in individuals carrying multiple minor alleles, may contribute to ischemic stroke risk stratification in patients with COVID-19 and to the determinants of classical thrombotic disorders, underscoring the importance of polygenic interaction in modulating stroke risk during COVID-19 infection. These findings indicate that ischemic stroke associated with COVID-19 has a distinct genetic background compared with ischemic stroke of other etiologies. In particular, MTHFR rs1801133 appears to function as a specific genetic marker for COVID-19-related ischemic stroke, whereas other folate cycle gene polymorphisms contribute to a general thrombophilic predisposition. From a clinical perspective, identification of high-risk genotypes, especially in individuals carrying multiple minor alleles, may enable personalized risk stratification, targeted prevention, and early intervention in patients with COVID-19 [23,24,25].

A limitation of this study is the absence of detailed adjustment for COVID-19 severity markers, such as ICU admission, oxygen requirement, and inflammatory biomarkers. Further large-scale and prospective studies are warranted to validate these findings and to clarify how genetic susceptibility interacts with inflammatory and coagulation pathways in COVID-19-associated cerebrovascular complications [26,27].

The results of the study showed that the MTHFR gene rs1801133 (C677T) polymorphism is a specific genetic marker for the development of COVID-19-associated ischemic stroke. While patients carrying the minor T allele had a 4.05-fold increased risk of developing ischemic stroke against the background of COVID-19 (95% CI: 1.94–8.42; *p* < 0.001), no such statistically significant association was observed in the development of COVID-19-independent ischemic stroke (*p* > 0.05). This confirms the syntropic effect of the MTHFR C677T polymorphisms, as well as biochemical parameters, and risk, allowing for early identification of a high-risk group. This approach has practical implications for improving ischemic stroke prevention and determining personalized therapeutic measures.

## 4. Materials and Methods

### 4.1. Study Design and Population

This clinical observational study was conducted between 2021 and 2023 at the Zangiota Infectious Diseases Hospitals. A total of 110 patients with laboratory-confirmed COVID-19 infection were enrolled. Patients were stratified into study groups according to the presence or absence of ischemic stroke. The experimental group (*n* = 60) comprised individuals who developed ischemic stroke either during the acute phase of COVID-19 infection or within three months following recovery (post-COVID period). Comparison group (*n* = 50): COVID-19 patients who did not develop ischemic stroke. Control group (*n* = 50): patients with a documented history of ischemic stroke prior to the COVID-19 pandemic, without COVID-19 infection, were included to eliminate the confounding effect of SARS-CoV-2. This design allowed assessment of: (1) whether genetic polymorphisms increase stroke risk in post-COVID patients, and whether COVID-19 amplifies the pathogenic effect of thrombophilia and cytokine gene variants.

Inclusion criteria: laboratory-confirmed SARS-CoV-2 infection (pcr and/or ELISA), age > 40 years, clinically and neuroimaging-verified ischemic stroke, and written informed consent for participation.

Exclusion criteria: absence of confirmed COVID-19 infection, hemorrhagic stroke or degenerative neurological diseases, severe comorbid conditions (e.g., heart failure, cirrhosis, and renal failure), and patients younger than 18 years. Clinical assessment and clinical evaluation: Anamnesis collection, demographics, lifestyle, comorbidities, COVID-19 vaccination status, and past medical history. Objective examination: general physical status, vital signs, BMI, and cardiovascular and respiratory assessment. COVID-19 severity classification: based on national “Interim Recommendations for the Treatment of Coronavirus Infection.” ELISA was used to detect SARS-CoV-2-specific IgM/IgG antibodies, while PCR tests identified active viral RNA. COVID-19 severity was categorized as mild, moderate, severe, or critical. A statistically significant association was observed between COVID-19 severity and ischemic stroke (severe and critical forms were predominantly seen in the experiment group; *p* < 0.05).

### 4.2. Molecular-Genetic Analysis

DNA extraction Venous blood (1 mL) was collected in anticoagulant tubes and stored at +4 °C. Genomic DNA was isolated using the PureLink Genomic DNA Mini Kit (Invitrogen, Thermo Fisher Scientific, Waltham, MA, USA). according to the manufacturer’s protocol, based on silica-membrane purification. DNA samples were eluted in 25–200 μL of elution buffer and stored at −20 °C. Genotyping Real-time PCR was performed in the “Genotechnology” laboratory using the DTlite PCR system (DNA-Technology LLC, Moscow, Russia). The following polymorphisms were analyzed: Folate cycle/thrombophilia genes: *MTHFR* rs1801133 (C677T), rs1801131 (A1298C), *MTR* rs1805087 (A2756G), and *MTRR* rs1801394 (A66G). PCR reactions contained primer-probe mixes, hot-start Taq polymerase, dNTPs, MgCl_2_, and optimized buffers. Amplification was conducted according to the protocol. Fluorescent detection used FAM and ROX channels, and allelic discrimination plots were generated automatically.

### 4.3. Statistical Analysis

Data processing was performed using Statistica 6.1 and GraphPad Prism 6. Quantitative variables were expressed as mean ± SD. Group comparisons used the Student’s *t*-test. Genotype and allele frequencies were tested for Hardy–Weinberg equilibrium. Associations between polymorphisms and stroke risk were evaluated using the Chi-square test (χ^2^), Odds ratio (OR), and relative risk (RR) with 95% confidence intervals. Multiple logistic regression assessed gene-gene interactions and their syntropic (combined) effects on disease risk. Statistical significance was set at *p* < 0.05.

## Figures and Tables

**Table 1:** (A) Diagnostic and prognostic performance of minor alleles of folate cycle gene polymorphisms. Sensitivity (SE), specificity (SP), area under the ROC curve (AUC), odds ratio (OR), 95% confidence interval (CI), and *p*-values for minor alleles of MTHFR C677T, MTHFR A1298C, MTR A2756G, and MTRR A66G were calculated by comparing the experimental and comparison groups with the control group. (B) Diagnostic and prognostic performance of genotypes of folate cycle gene polymorphisms. Sensitivity (SE), specificity (SP), AUC, OR, 95% CI, and *p*-values are shown for selected genotypes of MTHFR C677T, MTHFR A1298C, MTR A2756G, and MTRR A66G, based on comparisons between the experimental and comparison groups versus the control group. (C) Synergistic effect of multiple minor alleles of folate cycle gene polymorphisms. Sensitivity (SE), specificity (SP), AUC, OR, 95% CI, and *p*-values are presented for the combined presence of two or three minor alleles in the experimental and comparison groups.

**A. Indi** **cators of sensitivity, specificity and prognostic efficiency in the minor allele of polymorphisms examined in patient groups**								
**Polymorphism**	**Allele**	**Groups**	**SE**	**SP**	**AUC**	**OR**	**95%CI**	***p*** **-Value**
MTHFR gene C677T	T	Experiment gr/Control gr	0.33	0.89	0.586	4.05	1.94–8.42	<0.001
		Comparison gr/Control gr	0.18	0.89	0.535	1.76	0.792–3.98	0.16
MTHFR gene A1278C	C	Experiment gr/Control gr	0.22	0.92	0.54	3.33	1.44–7.73	0.004
		Comparison gr/Control gr	0.16	0.92	0.54	2.19	0.892–5.38	0.08
MTR gene A2756G	G	Experiment gr/Control gr	0.183	0.95	0.531	4.2	1.55–11.7	0.003
		Comparison gr/Control gr	0.22	0.95	0.585	5.36	1.94–14.80	0.008
MTRR gene A66G	G	Experiment gr/Control gr	0.11	0.92	0.477	1.41	0.55–3.52	0.47
		Comparison gr/Control gr	0.10	0.92	0.510	1.27	0.322–5.07	0.12
**B. Indicators of sensitivity, specificity and prognostic efficiency in the genotype of polymorphisms examined in patient groups**								
MTHFR gene C677T	TT	Experiment gr/ Control gr	0.1	0.98	0.50	5.4	0.63–46.8	0.08
		Comparison gr/Control gr	0.04	0.98	0.51	2.04	0.179–23.27	0.56
MTHFR gene A1278C	CC	Experiment gr/ Control gr	0.067	1.0	0.49	-	-	0.06
		Comparison gr/Control gr	0.02	1.0	0.51	-	-	0.30
MTR gene A2756G	GG	Experiment gr/ Control gr	0.05	1.0	0.48	-	-	0.11
		Comparison gr/Control gr	0.04	1.0	0.52	-	-	0.15
MTRR gene A66G	GG	Experiment gr/ Control gr	0.02	1.0	0.46	-	-	0.36
		Comparison gr/Control gr	0.02	1.0	0.51	-	-	0.31
**C.** **Syntropic significance of minor alleles in folate cycle gene** **p** **olymorphisms in the development of ischemic stroke**								
**Allele-allele effect**	**Groups**	**SE**	**SP**	**AUC**	**OR**	**95%CI**		***p*** **-value**
Three times	Experiment group	0.08	1.0	0.50	-	-		0.03
	Comparison group	0.02	1.0	0.51	-	-		0.3
Twice	Experiment group	0.38	0.90	0.63	5.59	1.94–16.155		<0.001
	Comparison group	0.30	0.90	0.60	3.86	1.27–11.64		0.01

Abbreviation: SE, sensitivity; SP, specificity; AUC, area under the receiver operating characteristic curve; OR, odds ratio; and CI, confidence interval.

## Data Availability

The original contributions presented in this study are included in the article/Supplementary Materials. Further inquiries can be directed to the corresponding author(s).

## Supplementary Materials

The following supporting information can be downloaded at: https://www.mdpi.com/article/10.3390/ijms27062650/s1.
