# Supplementary material for: Thrombophilia and Folate Cycle Gene Polymorphisms in the Development of Ischemic Stroke After COVID-19
Source: Int J Mol Sci. 2026 Mar 13;27(6):2650. doi: 10.3390/ijms27062650 (PMC13026903; doi:10.3390/ijms27062650)
Supplement: Supplementary file 1 [file ijms-27-02650-s001.zip › ijms-4129051-supplementary.pdf]

Table S1. Folate cycle gene polymorphism frequency distribution in experiment and control groups.

Types of polymorism	Alleles and genotypes	Experiment group		Control group		OR	95%CI	χ2	p-value
		N	%	N	%				
MTHFR rs180113 3	C	80	66.7	89	89.0	0.25	0.12-0.51	15,3	<0,001
	T	40	33,3	11	11,0	4,05	1,94-8,42	15,3	<0,001
	CC	26	43,3	40	80,0	0,2	0,08-0,45	15,3	<0,001
	CT	28	46,7	9	18,0	3,99	1,65-9,63	10,0	0,002
	TT	6	0,1	1	2,0	5,4	0,63-46,8	2,9	0,08
MTHFR rs1801131	A	93	77,5	92	92,0	0,3	0,13-0,69	8,57	0,004
	C	27	22,5	8	8,0	3,33	1,44-7,73	8,57	0,004
	AA	37	61,6	42	84,0	0,31	0,122-0,77	6,72	0,01
	AC	19	31,6	8	16,0	2,43	0,96-6,17	3,6	0,058
	CC	4	6,7	0	0,0	-	-	3,45	0,06
MTR rs1805087	A	98	81,6	95	95,0	0,23	0,085-0,64	9,0	0,003
	G	22	18,3	5	5,0	4,2	1,55-11,7	9,0	0,003
	AA	41	68,3	45	90,0	0,24	0,08-0,70	7,51	0,007
	AG	16	26,7	5	10,0	3,27	1,1-9,70	4,9	0,027
	GG	3	5	0	0,0	-	-	2,57	0,11
MTRR rs1801394	A	107	89,2	92	92,0	0,72	0,28-1,80	0,51	0,47
	G	13	10,8	8	8,0	1,4	0,55-3,52	0,51	0,47
	AA	48	80	42	84,0	0,76	0,28-2,04	0,28	0,59
	AG	11	18,3	8	16.0	1,18	0.434-3.20	0,1	0,75
	GG	1	1,67	0	0,0	-	-	0,84	0,36

Table S2. Folate cycle gene polymorphism frequency distribution in the experiment and comparison groups.

Types of polymorism	Alleles and genotypes	Experiment group		Comparison group		OR	95%CI	χ2	p-value
		N	%	N	%				
MTHFR rs180113 3	C	80	66.7	82	82	0.44	0.23-0.83	6.6	0.01
	T	40	33.3	18	18	2.27	1.21-4.30	6.6	0.01
	CC	26	43.3	34	68	0 . 36	0.16-0.79	6.69	0.01
	CT	28	46.7	14	28	2.1	0.95-4.74	4.0	0.045
	TT	6	0.1	2	4	2.67	0.51-13.8	1.45	0.28
MTHFR rs1801131	A	93	77.5	84	84	0.66	0.33-1.30	1.46	0.23
	C	27	22.5	16	16	1.5	0.77-3.02	1.46	0.23
	AA	37	61.6	35	70	0.69	0.31-1.53	0.84	0.36
	AC	19	31.6	14	28	1.19	0.52-2.71	0.355	0.55
	CC	4	6.7	1	2	3.5	0.38-32.3	1.37	0.24
MTR rs 1805087	A	98	81.6	78	78	1.25	0.65-2.43	0.46	0.499
	G	22	18.3	22	22	0.80	0.41-1.54	0.46	0.499
	AA	41	68.3	30	60	1.44	0.65-3.15	0.83	0.363
	AG	16	26.7	18	36	0.64	0.287-1.45	1.11	0.292
	GG	3	5	2	4	1.26	0.20-7.87	0.06	0.8
MTRR rs1801394	A	107	89.2	90	90	0.91	0.38-2.18	0.04	0.84
	G	13	10.8	10	10	1.09	0.45-2.61	0.04	0.84
	AA	48	80	41	82	0.88	0.336-2.29	0.07	0.79
	AG	11	18.3	8	16	1.46	0.54-3.93	0.1	0.75
	GG	1	1.67	1	2	0.83	0.05-13.6	0.02	0.9
